# Characterization of a New Bifunctional and Cold-Adapted Polysaccharide Lyase (PL) Family 7 Alginate Lyase from *Flavobacterium* sp.

**DOI:** 10.3390/md18080388

**Published:** 2020-07-26

**Authors:** Hai-Xiang Zhou, Shan-Shan Xu, Xue-Jing Yin, Feng-Long Wang, Yang Li

**Affiliations:** 1 Tobacco Research Institute, Chinese Academy of Agricultural Sciences, Qingdao 266101, China; pro.zhouhaixiang@163.com; 2 College of Life Sciences and Oceanography, Shenzhen University, Shenzhen 518060, China; xushanshan328@163.com; 3 Qingdao Mental Health Center, Qingdao University, Qingdao 266034, China; yinxuejing@163.com; 4 School of Biology and Biological Engineering, South China University of Technology, Guangzhou 510006, China

**Keywords:** alginate lyase, bifunctional, cold-adapted, *Flavobacterium* sp. S02, *Yarrowia lipolytica*

## Abstract

Alginate oligosaccharides produced by enzymatic degradation show versatile physiological functions and biological activities. In this study, a new alginate lyase encoding gene *alyS02* from *Flavobacterium* sp. S02 was recombinantly expressed at a high level in *Yarrowia lipolytica*, with the highest extracellular activity in the supernatant reaching 36.8 ± 2.1 U/mL. AlyS02 was classified in the polysaccharide lyase (PL) family 7. The optimal reaction temperature and pH of this enzyme were 30 °C and 7.6, respectively, indicating that AlyS02 is a cold-adapted enzyme. Interestingly, AlyS02 contained more than 90% enzyme activity at 25 °C, higher than other cold-adapted enzymes. Moreover, AlyS02 is a bifunctional alginate lyase that degrades both polyG and polyM, producing di- and trisaccharides from alginate. These findings suggest that AlyS02 would be a potent tool for the industrial applications.

## 1. Introduction

Over the past decades, development and application of algal biomass have been increasingly emphasized in various fields [1,2,3]. As the major ingredient in the cell wall and intracellular matrix, alginate is the largest amount of polysaccharide in brown algae, accounting for as much as 40% of the dry weight [4]. Hence, it has become essential source of raw material nowadays. Alginate is a linear hetero-polysaccharide composed of β-D-mannuronate (M) and α-L-guluronate (G) units, which are linked by 1, 4-o-glycoside bonds and arranged into three kinds of block structures: polyM block, polyG block and alternating or random MG block (polyMG) [5]. Alginate has been used as a food additive thanks to its gelling property, high viscosity and chemical stability [6]. Besides, it has many other applications in pharmaceutical and food industries, chemical processes and biotechnology research [7,8,9,10]. Alginate oligosaccharides, the depolymerization products of alginate by enzymatic catalysis, have attracted more attention due to their better solubility and remarkable biological activities compared with macromolecular alginates [11]. They can be used as therapeutic agents such as anticoagulants, tumor inhibitors, anti-allergy medicine, and anti-viral drugs [12,13,14,15]. They also possess other diverse physiological functions that are beneficial to humans, e.g., antioxidation, regulating immune functions, and proliferating bifidobacteria [16,17,18,19]. In addition, alginate oligosaccharides can be used to stimulate protection against pathogens and as growth promoters for plants [20,21,22]. Therefore, the production of alginate oligosaccharides is extremely important for various fields.

Alginate lyase belongs to polysaccharide lyase (PL) family that cleaves the 1, 4-o-glycosidic bonds of alginate through β-elimination mechanism, thus creating a saturated uronate at the new reducing terminus and an unsaturated 4-deoxy-L-erythro-hex-4-enopyranosyluronic acid (Δ) with a double bond between C-4 and C-5 at the new non-reducing terminus [23]. Based on the action mode on substrates, alginate lyases are generally divided into endolytic and exolytic types [24]. Most alginate lyases exhibit endolytic activity that cleave internal glycosidic linkages of the alginate chain to release a mixture of unsaturated oligosaccharides with different degrees of polymerization (DPs) as major products [25,26,27,28,29]. However, there are also a few exo-type alginate lyases (EC 4.2.2.26), also known as oligoalginate lyases, which cut the glycoside bond at the end of the alginate polymer, generating products with homogeneous DP [30,31]. Moreover, endo-alginate lyases can be further classified into two groups in terms of the substrate specificities, i.e., polyguluronate-specific lyase (polyG lyase, EC4.2.2.11) which prefer catalyzing the degradation of polyG to polyM [32,33], and polymannuronate-specific lyase (polyM lyase, EC4.2.2.3) that display higher activities toward polyM [34,35]. But some enzymes manifest high activities against both polyG and polyM, called bifunctional alginate lysaes, which may degrade alginate more effectively [36,37,38]. Additionally, there still exist a special group among all the alginate lysaes that prefer breaking up MG-blocks to homogenous substrates, namely polyMG-specific lyases [39]. According to the Carbohydrate-Active Enzyme (CAZy) databases (http://www.cazy.org/fam/acc_PL.html), the reported alginate lyases have been organized into PL5, 6, 7, 14, 15, 17, and 18 families on the basis of their primary structures, whereas most of the enzymes belong to the PL7 family [40,41]. So far, alginate lyases, especially endo-type enzymes, have been not only applied in the preparation of alginate oligosaccharides [11,42,43], but also for biotechnology research (e.g., elucidation of alginate structures, preparation of protoplast) [44,45], disease treatment (e.g., therapy of cystic fibrosis, inhibition of pathogen bacteria) [46,47], and marine environmental protection (e.g., disposal of seaweed wastes) [48]. As a result, it is of great urgency to discover novel alginate lyases with excellent characteristics (i.e., high activity, high stability and broad substrate specificity).

Alginate lyases mainly distribute in diverse marine-derived organisms including microbes [33,49,50,51], mollusks [52], algae [53], and some *Chlorella* viruses [54]. In the past several years, many alginate lyases with various properties have been isolated, cloned, purified and characterized from those sources especially from a diversity of marine bacteria [55]. However, few of these reported enzymes have been commercially used owing to their poor properties [56]. Several alginate lyases with certain specific characteristics have been found and studied, such as pH stability [51,57], thermo-tolerance [58], cold adaption [59,60], acidic or alkaline adaption [61,62], single product distribution [63,64], which are quite significant for both research and commercial purposes, but the enzymes with optimal characteristics are rare. It is worth noting that cold-adapted alginate lyases make running biocatalysis at low temperature possible, which is crucial to reduce the danger of contamination and improve the sustainability of enzyme utilization, thereby decreasing the input costs to some extent, particularly for industrial production of oligosaccharides.

In this study, a novel bifunctional and cold-adapted PL family 7 alginate lyase AlyS02 with considerable properties was purified and characterized in detail, after the encoding gene was identified in a marine-originated bacterium *Flavobacterium* sp. S02 and expressed using *Yarrowia lipolytica*. The AlyS02 degraded alginate, producing di- and trisaccharides as major products. These special features suggest that AlyS02 would be a useful enzyme for industrial applications.

## 2. Results and Discussions

### 2.1. Sequence Analysis of AlyS02

**Figure 1:**
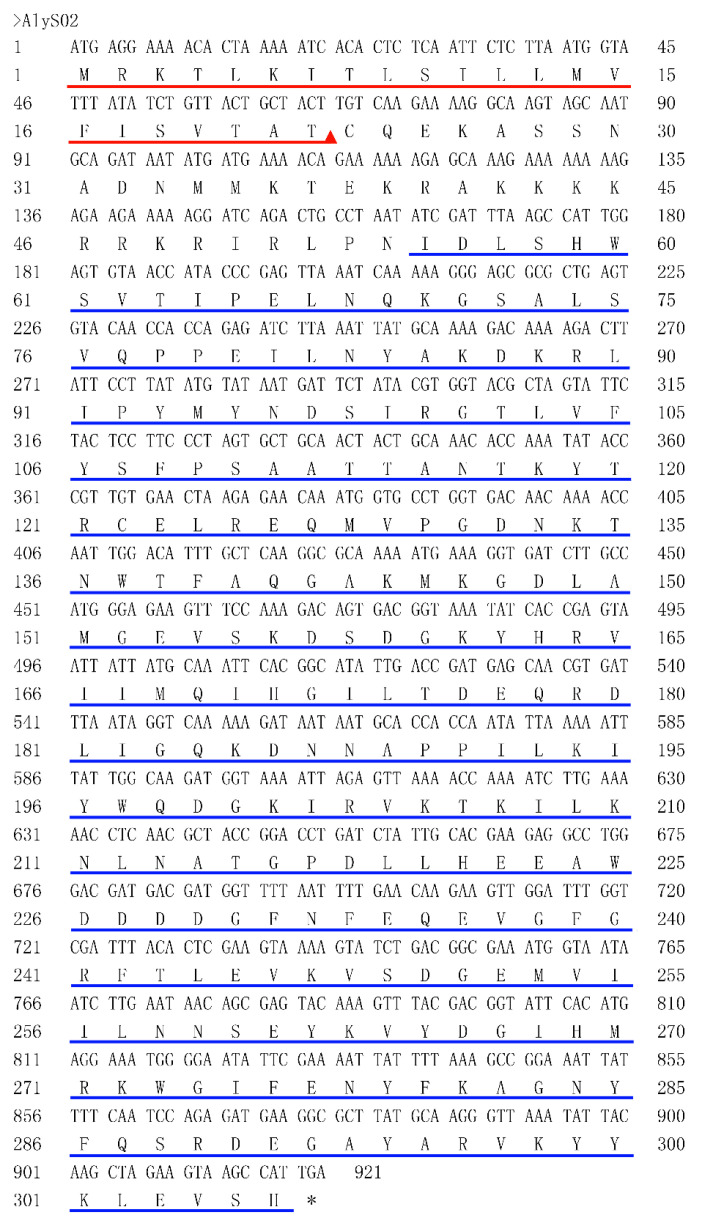
Nucleotide and its deduced amino acid sequences of alginate lyase gene *alyS02*. The ORF (open reading frame) was composed of 921 bp and encoded AlyS02 containing 306 amino acids with a putative signal peptide marked with a red line, and the predicted cleavage site between TAT-CQ. The alginate_lyase2 domain is underlined with a blue line.

The bacterium *Flavobacterium* sp. S02 was isolated from brown seaweed in the Yellow Sea, China, the genomic analysis of which implied there existed a putative alginate lyase-encoding gene *alyS02* (Genbank number MT338519), with 921 bp of open reading frame (ORF). Thus, the translated AlyS02 consisted of 306 amino acid residues with a putative signal peptide (Met^1^ to Thr^22^) in its N-terminal predicted by signal peptide analysis (Figure 1). Furthermore, the molecular weight (Mw) and pI value of the mature enzyme were calculated as 32.79 kDa and 9.11, respectively. According to the analysis of the InterProScan application, a single-domain (Ile^55^-His^306^) that belongs to alginate lyase superfamily 2 was identified in the novel alginate lyase AlyS02 (Figure 1).

**Figure 2:**
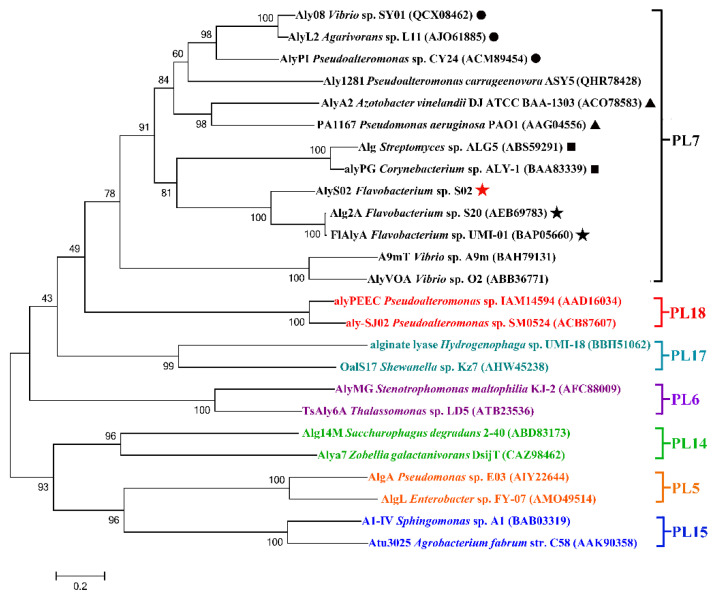
The phylogenetic analysis of AlyS02 and related alginate lyases. Bar, 0.2 substitutions per nucleotide position. The red five-pointed star indicates AlyS02 in this study. Different families are shown in different colors. In the PL7 family, black triangles, solid circles, boxes and five-pointed stars indicate subfamily 1, subfamily 3, subfamily 5 and subfamily 6, respectively.

On the basis of the sequences of AlyS02 and some other alginate lyases reported from different families, a phylogenetic tree was created through the Neighborhood-joining method (Figure 2), indicating that AlyS02 belonged to PL family 7. Moreover, the alginate lyases from that family can be grouped into several subfamilies. As displayed in Figure 2, AlyS02 (Genbank number MT338519) was at the same deeply branched cluster with Alg2A (Genbank number AEB69783) and FlAlyA (Genbank number BAP05660), demonstrating that AlyS02 was a member of subfamily 6.

**Figure 3:**
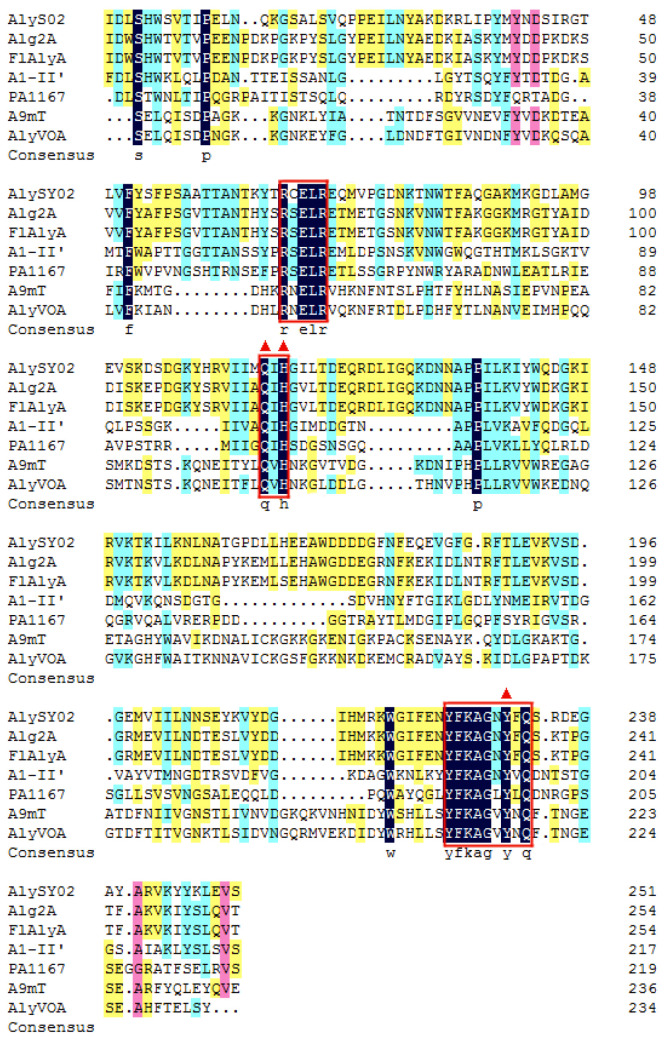
Multiple sequence alignment of A1yS02. AlyS02 from *Flavobacterium* sp. S02 in this study, Alg2A (AEB69783) from *Flavobacterium* sp. S20, FlAlyA (BAP05660) from *Flavobacterium* sp. UMI-01, A1-II’ (BAD16656) from *Sphingomonas* sp. A1, PA1167 (AAG04556) from *Pseudomonas aeruginosa* PAO1, A9mT (BAH79131) from *Vibrio* sp. JAM-A9m, AlyVOA (ABB36771) from *Vibrio* sp. O2. The conserved regions are highlighted with red boxes. Red triangles indicate the potential residues involved in the catalytic activity in the PL7 family.

Among all the reported alginate lyases, AlyS02 was most closely related to alginate lyase FlAlyA (GenBank number BAP05660) from *Flavobacterium* sp. UMI-01 and Alg2A (GenBank number AEB69783) from *Flavobacterium* sp. S20 with the sequence identities of both 56.31%. The alignment of the alginate_lyase2 domains among AlyS02 and several previously characterized alginate lyases from PL7 are shown in Figure 3, indicating that AlyS02 contained 3 highly conserved regions of RXELR, Q(I/V)H and YFKAGXYXQ, which were responsible for binding and catalyzing the substrate in PL7 family. It has been reported that QIH and QVH are related to substrate recognition, the former recognizes polyG and polyMG [61], such as Alg2A from *Flavobacterium* sp. S20 (Figure 3), and the latter prefers polyM [65], implying that AlyS02 may be polyG-preferred. Moreover, catalytic sites (Q169, H171, and Y285) were predicted in AlyS02 based on previous research on alginate lyase AlyA5 from *Zobellia galactanivorans* DsijT and Aly1281 from *Pseudoalteromonas carrageenovora* ASY5 [64,66]. The results of sequence alignment further confirmed that AlyS02 belongs to the PL7 family.

### 2.2. Expression and Purification of Recombinant AlyS02

The vector pINA1312-*alyS02* was successfully constructed in *Escherichia coli* DH5α and transformed into *Y. lipolytica* after linearization. The transformant Y32 possessed the highest extracellular activity (36.8 ± 2.1 U/mL). After 72 h cultivation at 30 °C in GPPB medium, the recombinant AlyS02 was purified using Ni-NTA Sepharose affinity chromatography, eventually reaching 442.84 U/mg (Table 1). The specific activity of recombinant AlyS02 was subject to a slight decrease after the second ultrafiltration step, which might be caused by the immense pressure coming from the collisions between protein molecules and ultrafiltration membrane during the high-speed centrifugation. In this work, engineering strain *Y. lipolytica* URA^−^ was used to express the recombinant AlyS02 rather than other commercial stains such as *E. coli* BL21 or *Pichia pastoris* X33, firstly because of the insecurity of the strains above (e.g., methanol used as inducer for *P. pastoris*) [67], whereas the yeast *Y. lipolytica* has been identified by FDA as GRAS (generally regarded as safe). Not only that, due to the remarkable capability of post-translational processing and secreting proteins, *Y. lipolytica* is recognized as a favorable choice for heterologous protein expression [68].

**Figure 4:**
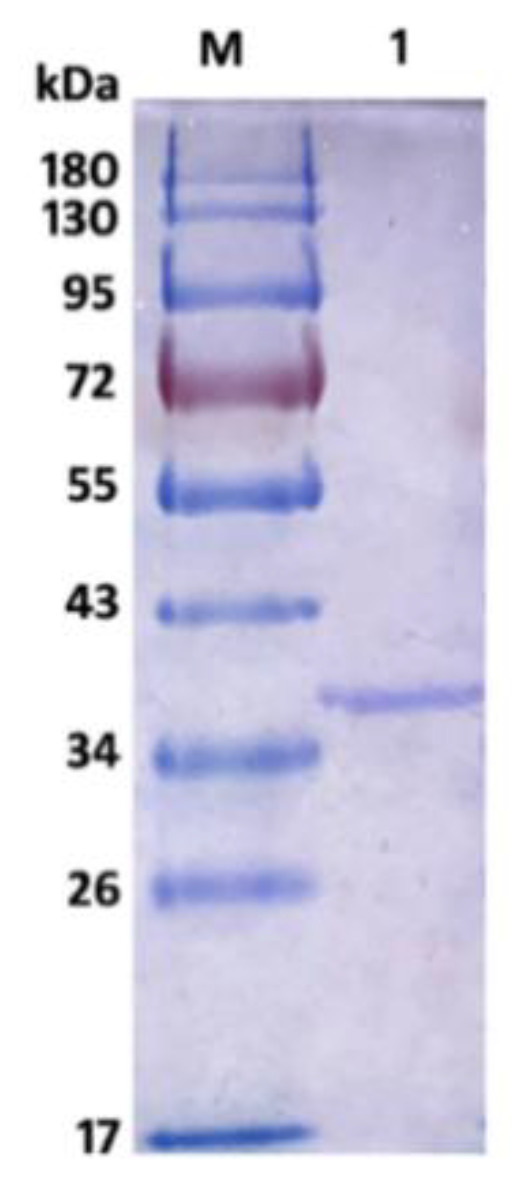
SDS-PAGE of purified AlyS02. Lane M, protein marker; lane 1, purified AlyS02.

The recombinant AlyS02 was further analyzed by SDS-PAGE, and the result is shown in Figure 4. There was a single band of recombinant Alys02 located at the gel with an Mw of approximate 36.5 kDa, suitable for downstream biochemical characterization. The Mw of AlyS02 was slightly higher than the theoretical value probably due to the recombinant AlySY02 fused with 6×His-tag, whose molecular mass would be larger with 312 amino acids. Moreover, recombinant AlyS02 might be glycosylated during expression and secretion proceeds by yeast [67]. The enzymes from diverse microorganisms differ in size, ranging from 23 to 110 kDa [11]. Depending on molecular masses, the alginate lyases have been classified into three groups: large (>60 kDa), medium (more or less 40 kDa) and small (25–30 kDa) [11,36]. Therefore, AlyS02 in this research belongs to the small-sized alginate lyases. The alginate lyases Cel32 from *Cellulophaga* sp. NJ-1 [11], alySJ-02 from *Pseudoalteromonas* sp. SM0524 [36], A1m from *Agarivorans* sp. JAM-A1m [62], and the alginate lyase from *Pseudoalteromonas* sp. strain No. 272 [69], have molecular masses of 32 kDa, 32 kDa, 31 kDa and 33.9 kDa, respectively, similar to the Mw of AlyS02.

### 2.3. Characterization of Recombinant AlyS02

**Figure 5:**
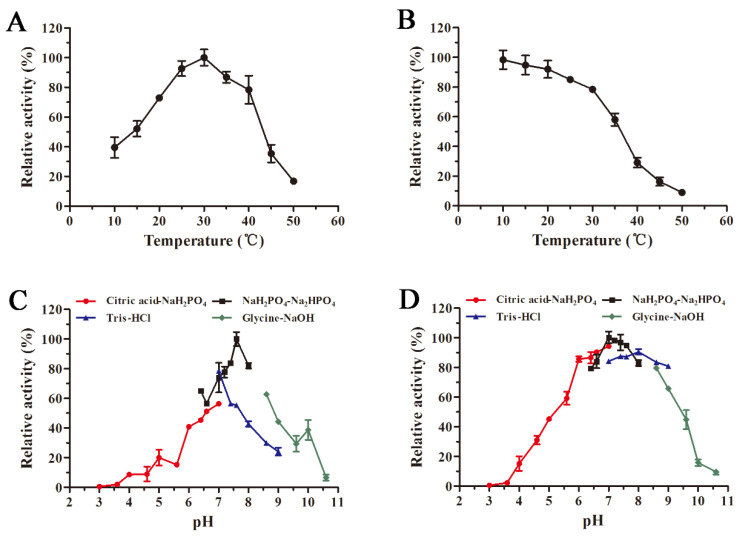
Influences of temperature and pH on the activity and stability of AlyS02. (**A**) Optimal temperature of AlyS02. The enzyme activities were measured at different temperatures (10–50 °C) and the relative activities were calculated by setting the activity at the optimum temperature as 100%. (**B**) Thermal stability of AlyS02. The residual activities were tested after incubation for 2 h at different temperatures (10–50 °C) and calculated by using the initial activity as 100%. (**C**) Optimal pH of AlyS02. The enzyme activities were determined with the citric acid-NaH_2_PO_4_ buffer (pH 3.0–7.0), NaH_2_PO_4_-Na_2_HPO_4_ buffer (pH 6.4–8.0), Tris-HCl buffer (pH 7.0–9.0), and Glycine-NaOH buffer (pH 8.6–10.6), and the relative enzyme activities were calculated by taking the activity at optimal pH as 100%. (**D**) pH stability of AlyS02. The residual activities were detected after AlyS02 was incubated for 24 h at 4 °C in the pH 3.0–10.6 in the buffers above, and the maximum residual activity was considered as 100%. Relative activity is expressed as a percentage and the data represented as the mean ± standard deviation of triplicate measurements.

The recombinant AlyS02 presented the optimal temperature of 30 °C and contained more than 70% of relative activity in the range of 20–40 °C (Figure 5A). Compared with other enzymes, AlyS02 had a wider temperature range for the activity. However, the activity of AlyS02 significantly reduced when the temperature exceeded 40 °C (Figure 5A). These results demonstrated that AlyS02 was a cold-adapted enzyme. Similar to AlyS02, the optimum temperatures of most cold-adapted alginate lyases reported are 30–35 °C, as displayed in Table 2. However, AlyS02 still possessed more than 90% of maximum activity at 25 °C, higher than other cold-adapted enzymes at the same condition, such as AlyPM from *Pseudoalteromonas* sp. SM0524 and TsAly6A from *Thalassomonas* sp. LD5 [59,60], meaning that AlyS02 could be used at room temperature which is convenient and conducive to cost reduction. AlyS02 remained around 80% activity after incubation for 2 h at 30 °C, and with favorable thermostability at the temperature below 30 °C (Figure 5B). Combined with relatively high activity at room temperature, AlyS02 was able to degrade the alginate completely, releasing oligosaccharides before its inactivation. However, the thermostability of AlyS02 declined significantly when the temperature came to be higher than 30 °C, signifying it should be stored for a long time at a lower temperature. A considerable advantage of expression proteins in eukaryotic cells is that diverse modifications could be introduced during post-translational processing, among which the glycosylation has been found with the function to enhance the thermostability of proteins [70]. Thus, the approach of improvement of the stability of the enzymes by adding extra N-glycosylation sites in the protein molecules via site-directed mutagenesis was advisable [71]. Enzyme immobilization is another effective means to further stabilize enzymes. Kunjukunju et al. prepared a cross-linked aggregate of alginate lyase that was thermally stable up to 50 °C over a period of 8 h, and compared with the native enzyme the reusability and storage stability were both improved [72]. The favorable methods described above would be used in the future for stabilizing alginate lyases, especially cold-adapted ones such as AlyS02.

The optimal pH of recombinant AlyS02 was determined as 7.6 and it maintained over 60% of relative activity at pH ranging from 6.4 to 8.6 (Figure 5C). AlyS02 was mostly stable at pH 7.0 and had a favorable stability at a broad pH range of 6–9, with over 80% activity retained after being incubated for 24 h (Figure 5D). Therefore, AlyS02 was an alkaline-stable enzyme. Generally, the alginate lyases from PL7 family preferred neutral or slightly alkaline conditions. As shown in Table 2, pH 7–8.5 is the generally optimal pH characteristic of the alginate lyases from the PL7 family, except Aly2 from *Flammeovirga* sp. MY04, whose optimal pH was 6.0 [73]. Surprisingly, under the same pH conditions, the enzyme activity with Tris-HCl buffer was significantly lower than that in phosphate buffer (Figure 5C), which has been reported before [27,69]. The possible reasons for this phenomenon are that AlyS02 was activated by Na^+^ and Tris would destroy the enzyme more or less.

**Figure 6:**
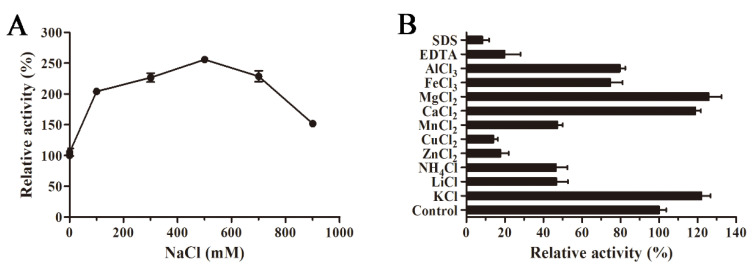
Influences of NaCl, various metal ions and chemical compounds on AlyS02 activity. (**A**) Effect of NaCl on AlyS02. The activity of the control was relatively taken as 100%. (**B**) Effect of different metal ions, EDTA and SDS on AlyS02. Each value represents the mean of three replicates ± standard deviation.

The influence of NaCl concentration on AlyS02 activity is revealed in Figure 6A. AlyS02 was activated within 100–900 mM of NaCl, and the activity reached the maximum with 500 mM NaCl, approximately 250% of relative activity compared to that without NaCl (Figure 6A). The results indicated that AlyS02 was a salt-activated and salt-tolerant alginate lyase, but not dependent on Na^+^. This phenomenon also partially explained the influences of various buffer solutions in the tests of pH characteristics. The salt-activated property was common in the enzymes isolated from marine bacteria (Table 2), where the survival of these strains and activation of the enzymes need the environment to have a certain degree of NaCl concentration. The effects of various metals (1 mM) on AlyS02 activity were also examined. As exhibited in Figure 6B, the enzyme activity was increased with Mg^2+^, Ca^2+^, and K^+^, just as most alginate lyases listed in Table 2. However, the enzyme activity was prominently reduced with SDS, EDTA, Cu^2+^ and Zn^2+^, and partially decreased with other metal ions such as ${NH}_{4}^{+}$, Li^+^, Mn^2+^, Al^3+^ and Fe^3+^. Similar situations could be found in many alginate lyases originated from marine environments; most trivalent and divalent heavy metal ions and other reagents such as SDS and EDTA can significantly inhibit the activities of the enzymes, perhaps owing to the denaturation of enzymes (Table 2). However, some exceptional cases exist in previous studies. Interestingly, SDS could activate AlyYKW-34 from *Vibrio* sp. YKW-34 [75]. The activity of Aly2 from *Flammeovirga* sp. MY04 would be enhanced in the presence of EDTA [73], and Ba^2+^ and Co^2+^ showed obviously activating effects on aly-SJ02 from *Pseudoalteromonas* sp. SM0524 [36]. The influences of ions on the enzymes derived from diverse species may be different due to the various environments for diverse bacteria strains to survive and evolve [25].

### 2.4. Substrate Specificity and Degradation Pattern of AlyS02

**Figure 7:**
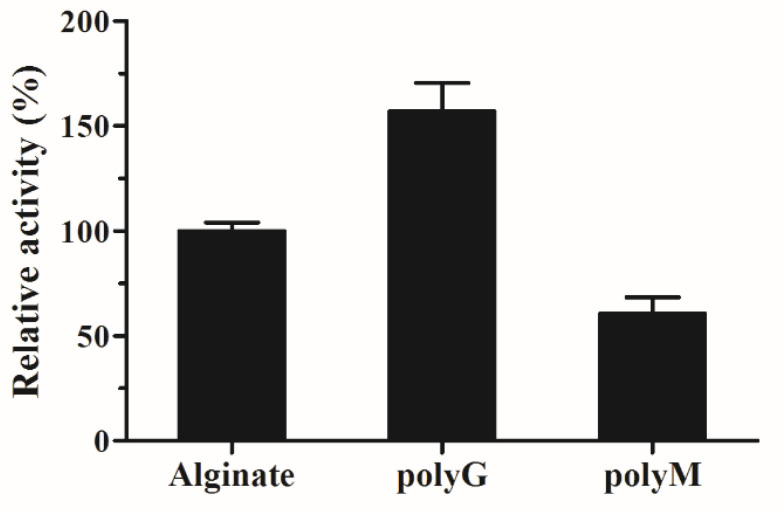
Substrate specificity of AlyS02. Substrates were alginate, poly G and polyM.

To investigate the substrate specificity of AlyS02, three kinds of polymeric substrates (polyG, polyM and sodium alginate) were utilized. The relative catalytic activities of AlyS02 against tested substrates are shown in Figure 7. The result indicated that this enzyme is a bifunctional alginate lyase which degrades both polyM and polyG (Figure 7). Most alginate lyases are specific to M block or G block, called monofunctional enzymes [33,35,61,74]. The utilization of those enzymes was limited except for some particular purpose such as preparation of polyM block from alginate through degrading the polyG and polyMG blocks [76]. However, AlyS02 with broader substrate specificity was capable of degrading alginate more effectively, similar to other bifunctional alginate lyases reported [11,19,29,36,37]. The Q(I/V)H conserved sequences important for substrate recognition and catalysis exist in almost all the alginate lyases from PL7 family [25,55]. In the current study, AlyS02 was found to degrade polyG more efficiently than the other assayed polymeric substrates (Figure 7); this result corresponded with the QIH sequence in the primary structure of AlyS02 described previously. Nevertheless, there still are some exceptions to this rule. Yagi et al. reported that rAlgSV1-PL7 with QVH was highly active against polyG [27]. The polyM-preferred FlAlyA contains QIH in the amino acid sequence [26].

**Figure 8:**
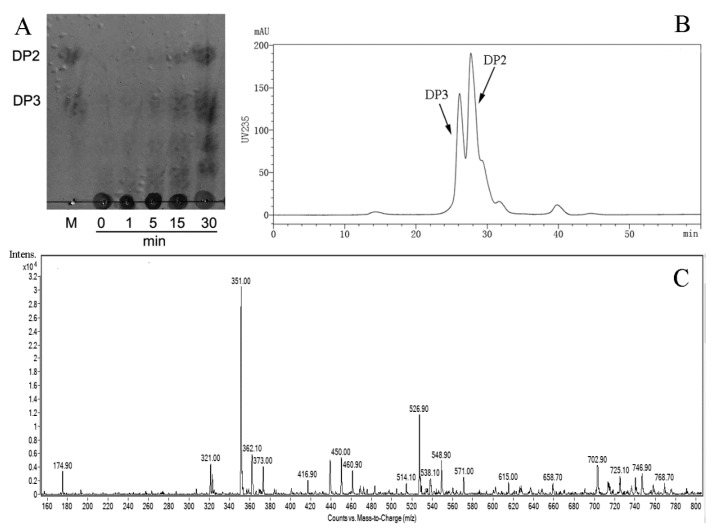
The products generated by AlyS02-degrading alginate were analyzed by TLC (thin layer chromatography), SEC (size exclusion chromatography) and ESI-MS (electrospray ionization mass spectrometry). (**A**) TLC analysis of the depolymerization products of AlyS02 toward alginate for 0, 1, 5, 15 and 30 min. DP2 indicates dimer, DP3 indicates trimer. (**B**) SEC analysis of the degradation products of AlyS02 toward alginate. (**C**) ESI-MS analysis of the degradation products of AlyS02 against alginate.

In order to further determine the pattern of AlyS02 action towards sodium alginate, thin layer chromatography (TLC), size exclusion chromatography (SEC) and electrospray ionization mass spectrometry (ESI-MS) methods were used to analyze the degradation products (Figure 8). The process of catalyzed breakdown was analyzed firstly by TLC assay at different time intervals. As shown in Figure 8A, with gradual reduction of alginate polysaccharides, the oligosaccharides with low DPs appeared and accumulated as the proceeding of enzymatic reaction. After incubation for 30 min, two clear spots whose migration rates were in line with the alginate disaccharide (DP2) and trisaccharide (DP3) marker on the TLC plate manifested that the hydrolysis pattern of AlyS02 was endo-type.

Furthermore, the distribution of final depolymerization products was monitored by SEC using high performance liquid chromatography (HPLC) platform, and dimers and trimers were proved to be the major products (Figure 8B). The result of ESI-MS is displayed in Figure 8C and DPs with the corresponding mass-to-charge ratios (*m*/*z*) of unsaturated oligosaccharides are shown in Table 3. Under the negative mode, DPs of the end product were implied to be 2 (351.0 *m*/*z*) and 3 (526.9 *m*/*z*) owing to the predominant ion peaks of [ΔDPx–H]^−^ (x = 2, 3) [19,42]. There were also some weak peaks consistent with DP 2, 3 of the products (Figure 8C and Table 3). It is necessary to mention that the ion peak with 174.9 *m*/*z* represented disaccharides from [ΔDP2–2H]^2–^ species rather than monosaccharides [29], owing to no monosaccharide was detected using TLC and SEC methods, which differ from the earlier researches of rAlgSV1-PL7 and Aly08 [27,61].

All the results illustrated that AlyS02 depolymerized alginate polymers in an endolytic manner into unsaturated disaccharides and trisaccharides eventually, between which the former accounted for a larger fraction. The findings were similar to other endo-alginate lyases from PL7 family in Table 2, which released the oligosaccharides of DP 2, 3 as main products too [11,61,73]. Although alginate lyases with a variety of action modes degrade alginate to produce oligosaccharides with different DPs, such as 2–4 [36,39], 2–5 [26,33] and 3, 4 [77], DP values of catalyzed products do not exceed 2–5 in most cases [40]. However, alginate lyases with special action patterns have also been discovered. Alg2A from *Flavobacterium* sp. S20 appears to take penta-, hexa- and hepta-alginate oligosaccharides as the major depolymerization products [32]. Some alginate lyases can cleave the substrate mainly releasing the products with single DP distribution [63,64]. During the enzymatic preparation of oligosaccharides, high degradation specificity will be conducive for the oligosaccharide purification and application. The end products of AlyS02 are di- and trisaccharides, which are propitious to further separation and industrial high-efficiency production.

Currently, the biological activities and physiological functions of oligosaccharides derived from enzymatic degradation of alginate are being studied. Unsaturated mannuronate and guluronate oligomers (M3-M6 and G3-G6) can induce the secretion of a variety of cytokines, e.g., monocyte chemoattractant protein-1 (MCP-1), granulocyte colony-stimulating factor (G-CSF) and tumor necrosis factor-α (TNFα) in the mouse macrophage cell line RAW264.7 [14]. Pentamers contained antitumor activity aimed at osteosarcoma cells, which results from their antioxidant and anti-inflammatory activities [78]. Alginate oligosaccharides with DP 2–4 taking guluronic acid at the reducing terminus showed the effective stimulation to the proliferation and migration of human endothelial cells [79]. Moreover, alginate oligosaccharides with DP 3–6 could promote root growth of lettuce seedlings [80]. The bifunctional AlyS02 has potential to be a potent tool for the effective generation of highly specific unsaturated oligosaccharides which have fascinated researchers due to wide food and pharmaceutical applications.

## 3. Materials and Methods

### 3.1. Materials, Strains and Culture Conditions

Sodium alginate (M/G ratio: 1.66; viscosity: 500 cP) from *Macrocystis pyrifera* was purchased from Qingdao Bright Moon Seaweed Group Co., Ltd. (Qingdao, China). D-polymannuronic acid (PolyM; M/G ratio 97.3/2.7, purity: 99%) and L-polyguluronic acid (polyG; M/G ratio 1.8/98.2, purity: 99%) were bought from Qingdao BZ Oligo Biotech Co., Ltd. (Qingdao, China). Standard alginate disaccharide and trisaccharide were also purchased from Qingdao BZ Oligo Biotech Co., Ltd.

Marine-derived bacterium *Flavobacterium* sp. S02 was isolated from brown seaweed in the Yellow Sea, China. *E. coli* DH5α was used for plasmid construction and amplification, cultured in Luria-Bertani (LB) medium with appropriate antibiotics (100 μg/mL ampicillin or 50 μg/mL kanamycin) at 37 °C. The uracil mutant strain *Y. lipolytica* URA^−^ and pINA1312 vector used for *alyS02* gene expression were provided by Prof. Zhenming Chi, Ocean University of China. The recombinant strains of *Y. lipolytica* were screened on a YNB plate, containing 10.0 g/L glucose, 1.7 g/L yeast nitrogen base, 5.0 g/L (NH_4_)_2_SO_4_ and 20.0 g/L agar [81]. The GPPB liquid medium was applied for recombinant alginate lyase production by the transformant obtained, containing 30.0 g/L glucose, 2.0 g/L yeast extract, 1.0 g/L (NH_4_)_2_SO_4_, 3.0 g/L K_2_HPO_4_, 2.0 g/L KH_2_PO_4_, 0.1 g/L MgSO_4_·7H_2_O, pH 6.8 [81].

### 3.2. Sequence Analysis of AlyS02

In previous work, the marine bacterium *Flavobacterium* sp. S02 displayed the ability to degrade alginate and grow in an alginate sole-carbon medium (data not shown). The genomic sequence analysis of *Flavobacterium* sp. S02 implied that there was a putative alginate lyase-encoding gene, named *alyS02* and deposited in Genbank database (Genbank number MT338519). The ORF was recognized by ORF finder (https://www.ncbi.nlm.nih.gov/orffinder/). The signal peptide cleavage site of AlyS02 was predicted through the SignalP 5.0 server (http://www.cbs.dtu.dk/services/SignalP/). The conserved domain of AlyS02 and its family were analyzed using the InterProScan application (http://www.ebi.ac.uk/interpro/search/sequence/). The theoretical pI and Mw of AlyS02 were calculated with the compute pI/Mw Tool (https://web.expasy.org/compute_pi/).

The bootstrapped phylogenetic tree was constructed via MEGA 6.0 by the neighbor-joining method [82], based on the amino acid sequences of related alginate lyases acquired from NCBI (https://www.ncbi.nlm.nih.gov/). The alignment of amino acid sequences was carried out using DNAMAN 6.0 (Lynnon Biosoft, USA).

### 3.3. Secretory Expression and Purification of Recombinant AlyS02

To express AlyS02 in *Y. lipolytica*, the *alyS02* gene without signal sequence and stop codon was optimized through elimination of the rare codons used in that yeast and the negative cis-acting elements such as mRNA destabilizing motifs, RNase splicing sites and repetitive element (Figure S1). Then the optimized gene with a C-terminal 6×His-tag and N-terminal XPR2 signal peptide was synthesized by Genscript Biotech Co., Ltd. (Nanjing, China). In addition, two restriction sites (*Bam*H I and *Sfi* I) were added at the ends of the gene. After the synthesized DNA was digested by *Bam*H I and *Sfi* I, the purified fragment was ligated into the corresponding sites of the vector pINA1312 with an uracil synthesis gene (*URA3*) to create the final recombinant plasmid pINA1312-*alyS02* (Figure S2) [81].

The pINA1312-*alyS02* fragment linearized by *Not* I was transformed into *Y. lipolytica* URA^−^ strain by LiAc method [83]. After 84 h incubation in GPPB medium at 30 °C, positive transformants were tested for alginate lyase activities. Finally, the recombinant strain Y32 showed the highest extracellular activity. The recombinant strain Y32 was fermented in the GPPB medium for 72 h. After centrifugation at 4 °C and 10,000× *g* for 5 min, the culture supernatant was collected. BCA protein assay kit (Solarbio, Beijing, China) was used to determine the total protein concentration and the alginate lyase activity was detected as described below. The crude enzyme solution was concentrated by ultrafiltration with a centrifugal filter 3 K device (3000 nominal molecular mass limit) (Millipore, Burlington, MA, USA) and then was loaded on Ni-Sepharose column (GE Healthcare, Uppsala, Sweden) equilibrated with 300 mM NaCl and 50 mM phosphate buffer (pH 7.0). Then, the column was washed with the same buffer containing 20 mM imidazole, and the 6×His-tagged alginate lyase was eluted with the same buffer containing a linear gradient of imidazole (50–500 mM). The active fractions were pooled, concentrated, and desalted, meanwhile, the buffer was replaced by 50 mM Tris-HCl (pH 7.0) with a Millipore centrifugal filter 3 K device for further enzyme characterization. The Mw of recombinant AlyS02 was determined by 12% sodium dodecyl sulfate-polyacrylamide gel electrophoresis (SDS-PAGE) system (Bio-Rad, Hercules, CA, USA) and the loading amount of recombinant AlyS02 was 10 μL. The protein marker was purchased from Solarbio Life Sciences (Beijing, China).

### 3.4. Enzymatic Activity Assay of the Alginate Lyase

To determine the activity of recombinant enzyme, 100 μL of appropriately diluted AlyS02 was mixed with 900 μL of substrate solution (1% (*w*/*v*) sodium alginate, 20 mM Tris-HCl buffer, pH 7.6), and incubated for 10 min at 30 °C. The solution was heated in boiling water for 10 min to terminate the reaction. Then, 1 mL of 3, 5-dinitrosalicylate (DNS) solution was added to the solution, and the mixture was reacted in a boiling water bath for 10 min. Finally, the absorbance was measured at 540 nm. One unit of alginate lyase activity was defined as the amount of enzyme required to release 1 μmol of reducing saccharides (glucose equivalent) per min.

### 3.5. Effects of Temperature and pH on Recombinant AlyS02 Activity and Stability

To study the effect of temperature on the enzyme activity, the activities of AlyS02 at different temperatures (10–50 °C) were measured, calculating the relative enzyme activity by setting the activity at the optimum temperature as 100%. In addition, to evaluate the influence of temperature on AlyS02 stability, the enzyme was incubated for 2 h at different temperatures (10–50 °C); at this time, residual enzyme activities were measured and calculated by using the initial activity as 100%.

Meanwhile, the effects of pH (3.0–10.6) on the purified enzyme were investigated. Alginate lyase activities in different buffers, including 20 mM citric acid-NaH_2_PO_4_ (pH 3.0–7.0), NaH_2_PO_4_-Na_2_HPO_4_ (pH 6.4–8.0), Tris-HCl (pH 7.0–9.0) and glycine-NaOH (pH 8.6–10.6), were measured to assess the influence of pH on AlyS02 activity, calculating the relative enzyme activity by taking the activity at optimal pH as 100%. In addition, to test the effect of pH on the stability of AlyS02, the enzyme was incubated at 4 °C for 24 h in different buffers above (pH 3.0–10.6); here, the residual enzyme activities were measured and the maximum residual activity was considered as 100%.

### 3.6. Effects of NaCl and Metal Ions on the Activity of Recombinant AlyS02

Enzyme reactions were performed in the presence of different concentrations of NaCl (100–900 mM) to evaluate the effect of NaCl on the activity of AlyS02. The enzyme activity in the absence of NaCl was used as control. To determine the influences of metal ions and chemical compounds on the enzyme activity, the residual enzyme activities of AlyS02 were analyzed after incubating for 24 h at 4 °C with the addition of different metal ions, EDTA and SDS at the concentration of 1 mM. The reaction mixture without any chemical compound and metal ion was used as control.

### 3.7. Substrate Specificity of Recombinant AlyS02

In this study, 1% (*w*/*v*) substrate solutions (20 mM Tris-HCl, pH 7.6) with three kinds of polymers (polyG, polyM and sodium alginate) were used to measure the enzyme activities by the DNS method described above to assess the preferred substrate of recombinant AlyS02.

### 3.8. Degradation Products Analysis of Recombinant AlyS02

In order to clarify the mode of action of AlyS02 against sodium alginate, 0.5% (*w*/*v*) sodium alginate was used as the substrate, and completely degraded with excess enzyme (5 U per mg of substrate) at 30 °C. The reaction mixture was continuously stirred and the degradation products were detected at regular time intervals. Then, the mixture solution was boiled for 10 min after incubation, and centrifuged at 12,000× *g* for 15 min to remove the unsolved materials. For removing proteins and other undegraded macromolecules, the supernatant was loaded on a centrifugal filter 3 K device (Millipore, Burlington, MA, USA). Then the filtrate was collected for detection.

First, the products were analyzed with a solvent system (1-butanol/acetic acid/water 2:1:1, *v*/*v*/*v*) using a TLC plate (TLC silica gel 60 F254, Merck KGaA, Darmstadt, Germany). After spraying with sulfuric acid/ethanol reagent (1:4, *v*/*v*), the TLC plate was heated at 80 °C for 30 min to visualize the spots. The mixture of disaccharide and trisaccharide was applied as a marker, and the total uronic acid concentration was 0.2% (*w*/*v*).

Next, a Superdex ^TM^ peptide 10/300 gel filtration column (GE Healthcare, Boston, MA, USA) was used for gel filtration chromatography analysis, and the complete degradation products were monitored at a wavelength of 235 nm by the ultraviolet detection system. The mobile phase was 0.2 M NH_4_HCO_3_ at a flow rate of 0.6 mL/min on an HPLC platform (LC-20A, Shimadzu, Japan). To further determine the composition of the end product, the filtrate was collected and mixed with methanol (1:1, *v*/*v*) and then quantitatively injected into an ESI-MS instrument (Bruker Esquire HCT, Billerica, MA, USA). The DPs of the enzymolysis products were profiled in negative-ion mode under the following conditions: calibration dynamics, 2; cone voltage, 20.00 V; capillary voltage, 4.00 kV; desolvation temperature, 350 °C; source temperature, 150 °C; desolvation gas flow, 500 L/h; and cone gas flow rate, 50 L/h; scan range, 100–1500 *m*/*z*.

## 4. Conclusions

In this work, a novel PL7 family alginate lyase AlyS02 derived from marine bacterium *Flavobacterium* sp. S02 was expressed, purified and characterized. The optimal temperature and pH of this enzyme were 30 °C and 7.6, respectively. AlyS02 was a cold-adapted enzyme with favorable stability. Moreover, AlyS02 exhibited bifunctional activity to release disaccharides and trisaccharides from alginate. These remarkable properties imply that AlyS02 would be a superior candidate for industrial applications.

## Figures and Tables

**Table 1:** Summary of the purification of AlyS02.

Purification Step	Total Protein (mg)	Total Activity (U)	Specific Activity (U/mg)	Purification Fold	Yield (%)
Crude enzyme	43.14	3680	85.3	1	100
Ultrafiltration 1st	38.32	3520.5	91.87	1.08	95.67
Ni-NTA sepharose	4.86	2217.9	456.36	5.35	60.27
Ultrafiltration 2nd	4.72	2090.2	442.84	5.19	56.8

**Table 2:** Comparison of the properties of AlyS02 with the related alginate lyases from various microorganisms.

Enzyme Name	Organism/Source	PL Family	Mw(kDa)	Optimal pH/Temperature (°C)	Cation Activators	Cation Inhibitors	Substrate Specificity	Main Products (DP)	References
AlyS02	*Flavobacterium* sp. S02/*Y. lipolytica*	7	36.5	7.6/30	Na^+^, K^+^, Ca^2+^, Mg^2+^	Fe^3+^, Al^3+^, Mn^2+^, Cu^2+^, Zn^2+^, ${NH}_{4}^{+}$, Li^+^, SDS, EDTA	PolyG, polyM	2, 3	This study
Alg2A	*Flavobacterium* sp. S20/*E. coli*	7	33	8.5/45	Na^+^, K^+^	Ca^2+^, Mg^2+^, Co^2+^, Cu^2+^, Zn^2+^, Mn^2+^, Fe^2+^	PolyG	5–7	[32]
FlAlyA	*Flavabacterium* sp. UMI-01/native	7	30	7.7/55	Na^+^, K^+^, Ca^2+^, Mg^2+^	Co^2+^, Ni^2+^	PolyM, polyMG	2–5	[26]
PA1167	*P. aeruginosa* PAO1/*E. coli*	7	25	8.5/40	N. D.	N. D.	PolyMG	2–4	[39]
Aly1281	*P. carrageenovora* ASY5/*E. coli*	7	40.65	8.0/50	Na^+^, K^+^	N. D.	PolyG, polyM	2	[64]
AlgNJ-04	*Vibrio* sp. NJ-04/*E. coli*	7	50.19	7.0/40	Na^+^, K+, Ca^2+^	Fe^2+^, Cu^2+^, Zn^2+^	PolyG, polyM	2–5	[51]
A9mT	*Vibrio* sp. JAM-A9m/*E. coli*	7	28	7.5/30	Na^+^, K^+^, Li^+^, Rb^+^, Cs^+^, ${NH}_{4}^{+}$, Tween 20, Nonidet P40	Cu^2+^, Zn^2+^, Ni^2+^, Co^2+^, Sr^2+^, SDS	PolyM	N. D.	[74]
Algb	*Vibrio* sp. W13/*E. coli*	7	54.12	8.0/30	Na^+^, Ca^2+^, Co^2+^, Fe^2+^	Cu^2+^, Zn^2+^, Mn^2+^, Ba^2+^	PolyMG, polyG, polyM	2–5	[25]
Aly08	*Vibrio* sp. SY01/*E. coli*	7	35	8.35/45	Na^+^, Ca^2+^, Mn^2+^, Co^2+^, Zn^2+^	SDS, EDTA	PolyG	2, 3	[61]
Aly510–64	*Vibrio* sp. 510–64/native	N. D.	34.6	7.5/35	Na^+^, K^+^, Ca^2+^, Mg^2+^, Li^+^	N. D.	PolyG, polyMG	3	[63]
AlySY08	*Vibrio* sp. SY08/native	N. D.	33	7.6/40	Na^+^, K^+^, Ca^2+^, Mg^2+^	Fe^3+^, Al^3+^, Mn^2+^, Cu^2+^, Zn^2+^, SDS, EDTA, 2-mercaptoethanol	PolyG, polyM	2	[29]
AlgA	*Bacillus* sp. Alg07/native	N. D.	60	7.5/40	Na^+^, Ca^2+^, Mn^2+^, Mg^2+^, Co^2+^	Fe^3+^, Fe^2+^, Cu^2+^, Al^3+^, Hg^2+^, Ba^2+^, EDTA	PolyM	2–4	[35]
Cel32	*Cellulophaga* sp. NJ-1/native	N. D.	32	8.0/50	Na^+^, K^+^, Ca^2+^, Mg^2+^	Fe^2+^, Cu^2+^, Zn^2+^, Co^2+^, Ni^2+^	PolyMG, polyG, polyM	2, 3	[11]
rSAGL	*Flavobacterium* sp. H63/*P. pastoris*	7	32	7.5/45	Na^+^, K^+^, Mg^2+^	Co^2+^, Cu^2+^, Zn^2+^, Mn^2+^, Ca^2+^, Ni^2+^, Fe^3+^, Ag^+^	PolyM	2–4	[67]
rAlgSV1-PL7	*Shewanella* sp. YH1/*E. coli*	7	33.216	8.0/45	Na^+^, K^+^, Mg^2+^	Cu^2+^, Fe^3+^, N-bromosuccinimide	PolyG, polyM, polyMG	1–4	[27]
Aly2	*Flammeovirga* sp. MY04/*E. coli*	7	60.58	6.0/40	EDTA, glycerol, 2-mercaptoethanol	Ag^+^, Hg^2+^, Pb^2+^, Fe^3+^, Zn^2+^, Ni^2+^, Fe^2+^, Cu^2+^, Cr^3+^, K^+^, Mn^2+^, SDS	PolyG, polyM	2, 3	[73]
TsAly6A	*Thalassomonas* sp. LD5/*E. coli*	6	83.9	8.0/35	Ca^2+^, Mg^2+^, Na^+^	EDTA, SDS	PolyG, polyM	2, 3	[60]
aly-SJ02	*Pseudoalteromonas* sp. SM0524/native	18	32	8.5/50	Na^+^, K^+^, Ba^2+^, Ca^2+^, Mg^2+^, Mn^2+^, Co^2+^, Sr^2+^, Ni^2+^	EDTA	PolyG, polyM	2–4	[36]
A1-IV’	*Sphingomonas* sp. A1/*E. coli*	15	90	8.5/50	N. D.	Cu^2+^, Zn^2+^, Hg^2+^, Co^2+^	PolyM, polyMG	2, 3	[50]
AlgA	*Pseudomonas* sp. E03/*E. coli*	5	40.4	8.0/30	Na^+^, K^+^, Ca^2+^, Mg^2+^, Zn^2+^, Ba^2+^, PMSF, DTT	Co^2+^, Cu^2+^, Mn^2+^, Fe^2+^, Triton X-100, Tween 20, EDTA, SDS, urea	PolyM	2–5	[34]

**Table 3:** ESI-MS analysis for the DPs (degrees of polymerization) of alginate oligomers.

Ion Mode	*m*/*z*
[ΔDP2–H]–	351
[ΔDP2–2H]2–	175
[ΔDP2–2H+Na]–	373
[ΔDP3–H]–	527
[ΔDP3–2H+Na]–	549
[ΔDP3–3H+2Na]–	571

## Supplementary Materials

The following are available online at https://www.mdpi.com/1660-3397/18/8/388/s1. Figure S1: Comparison between the original and the codon-optimized *alyS02* gene sequences without signal sequence and stop codon. The mutation sites introduced by codon optimization were highlighted in red, and the deduced amino acid sequence encoded by both the genes was shown below. Figure S2: Recombinant plasmid pINA1312-*alyS02* for expression of alginate lyase AlyS02.
