# Supplementary material for: Characterization of a New Bifunctional and Cold-Adapted Polysaccharide Lyase (PL) Family 7 Alginate Lyase from Flavobacterium sp
Source: Mar Drugs. 2020 Jul 26;18(8):388. doi: 10.3390/md18080388 (PMC7460543; doi:10.3390/md18080388)
Supplement: Supplementary file 1 [file marinedrugs-18-00388-s001.pdf]

Figure S1

Original	1	TGT CAA GAA AAG GCA AGT AGC AAT GCA GAT AAT ATG ATG AAA ACA GAA AAA AGA GCA AAG
Optimized	1	TGC CAG GAA AAG GCT TCT TCC AAC GCC GAC AAC ATG ATG AAG ACT GAG AAG CGG GCT AAA
Amino acid	1	C Q E K A S S N A D N M M K T E K R A K
Original	61	AAA AAA AAG AGA AGA AAA AGG ATC AGA CTG CCT AAT ATC GAT TTA AGC CAT TGG AGT GTA
Optimized	61	AAG AAG AAG CGA CGA AAA CGA ATC CGT CTG CCC AAC ATT GAC TTG TCT CAC TGG TCG GTG
Amino acid	21	K K K R R K R I R L P N I D L S H W S V
Original	121	ACC ATA CCC GAG TTA AAT CAA AAA GGG AGC GCG CTG AGT GTA CAA CCA CCA GAG ATC TTA
Optimized	121	ACC ATA CCG GAA CTT AAC CAG AAG GGA AGC GCT CTT AGT GTT CAG CCC CCT GAG ATT CTG
Amino acid	41	T I P E L N Q K G S A L S V Q P P E I L
Original	181	AAT TAT GCA AAA GAC AAA AGA CTT ATT CCT TAT ATG TAT AAT GAT TCT ATA CGT GGT ACG
Optimized	181	AAC TAC GCG AAA GAC AAG AGA CTC ATC CCC TAC ATG TAC AAC GAC TCA ATT CGA GGT ACG
Amino acid	61	N Y A K D K R L I P Y M Y N D S I R G T
Original	241	CTA GTA TTC TAC TCC TTC CCT AGT GCT GCA ACT ACT GCA AAC ACC AAA TAT ACC CGT TGT
Optimized	241	CTG GTG TTC TAC TCT TTC CCC TCA GCT GCG ACT ACA GCC AAC ACC AAG TAT ACG CGA TGT
Amino acid	81	L V F Y S F P S A A T T A N T K Y T R C
Original	301	GAA CTA AGA GAA CAA ATG GTG CCT GGT GAC AAC AAA ACC AAT TGG ACA TTT GCT CAA GGC
Optimized	301	GAG CTA AGA GAA CAG ATG GTG CCT GGA GAT AAC AAA ACC AAC TGG ACC TTT GCC CAG GGC
Amino acid	101	E L R E Q M V P G D N K T N W T F A Q G
Original	361	GCA AAA ATG AAA GGT GAT CTT GCC ATG GGA GAA GTT TCC AAA GAC AGT GAC GGT AAA TAT
Optimized	361	GCC AAG ATG AAG GGT GAT CTG GCC ATG GGA GAA GTT TCC AAG GAC TCC GAC GGA AAG TAC
Amino acid	121	A K M K G D L A M G E V S K D S D G K Y
Original	421	CAC CGA GTA ATT ATT ATG CAA ATT CAC GGC ATA TTG ACC GAT GAG CAA CGT GAT TTA ATA
Optimized	421	CAC CGA GTC ATC ATC ATG CAG ATC CAT GGC ATT CTG ACC GAC GAG CAA AGA GAT CTC ATT
Amino acid	141	H R V I I M Q I H G I L T D E Q R D L I
Original	481	GGT CAA AAA GAT AAT AAT GCA CCA CCA ATA TTA AAA ATT TAT TGG CAA GAT GGT AAA ATT
Optimized	481	GGC CAA AAG GAC AAT AAT GCT CCC CCT ATT TTG AAA ATC TAC TGG CAG GAC GGC AAG ATC
Amino acid	161	G Q K D N N A P P I L K I Y W Q D G K I
Original	541	AGA GTT AAA ACC AAA ATC TTG AAA AAC CTC AAC GCT ACC GGA CCT GAT CTA TTG CAC GAA
Optimized	541	CGG GTC AAG ACT AAG ATT CTC AAG AAC CTA AAT GCA ACT GGA CCA GAC CTG CTG CAT GAG
Amino acid	181	R V K T K I L K N L N A T G P D L L H E
Original	601	GAG GCC TGG GAC GAT GAC GAT GGT TTT AAT TTT GAA CAA GAA GTT GGA TTT GGT CGA TTT
Optimized	601	GAG GCC TGG GAC GAC GAT GAC GGT TTC AAC TTT GAG CAG GAG GTT GGT TTT GGC CGG TTC
Amino acid	201	E A W D D D D G F N F E Q E V G F G R F
Original	661	ACA CTC GAA GTA AAA GTA TCT GAC GGC GAA ATG GTA ATA ATC TTG AAT AAC AGC GAG TAC
Optimized	661	ACC CTG GAG GTC AAG GTA AGT GAT GGC GAG ATG GTT ATT ATT CTC AAC AAC TCG GAG TAC
Amino acid	221	T L E V K V S D G E M V I I L N N S E Y
Original	721	AAA GTT TAC GAC GGT ATT CAC ATG AGG AAA TGG GGA ATA TTC GAA AAT TAT TTT AAA GCC
Optimized	721	AAG GTC TAC GAT GGC ATC CAC ATG CGA AAG TGG GGT ATC TTC GAG AAC TAC TTC AAG GCC
Amino acid	241	K V Y D G I H M R K W G I F E N Y F K A
Original	781	GGA AAT TAT TTT CAA TCC AGA GAT GAA GGC GCT TAT GCA AGG GTT AAA TAT TAC AAG CTA
Optimized	781	GGC AAT TAT TTC CAG TCC AGG GAT GAG GGA GCC TAT GCT CGC GTG AAA TAC TAC AAG CTG
Amino acid	261	G N Y F Q S R D E G A Y A R V K Y Y K L
Original	841	GAA GTA AGC CAT
Optimized	841	GAA GTG AGC CAC
Amino acid	281	E V S H

Figure S1. Comparison between the original and the codon-optimized *alyS02* gene sequences without signal sequence and stop codon. The mutation sites introduced by codon optimization were highlighted in red, and the deduced amino acid sequence encoded by both the genes was shown below.

Figure S2

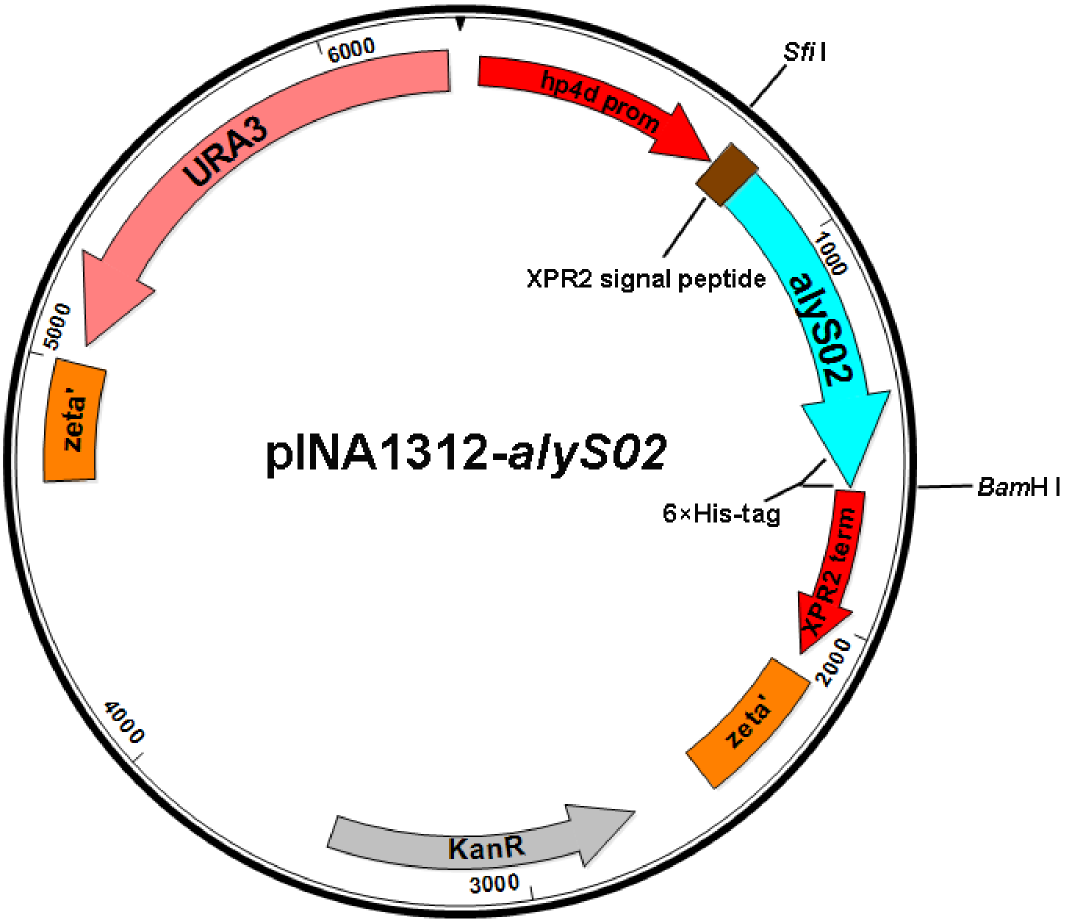

Figure S2. Recombinant plasmid pINA1312-*alyS02* for expression of alginate lyase AlyS02.
